# Impaired non-homologous end joining in human primary alveolar type II cells in emphysema

**DOI:** 10.1038/s41598-018-37000-z

**Published:** 2019-01-29

**Authors:** Beata Kosmider, Chih-Ru Lin, Liudmila Vlasenko, Nathaniel Marchetti, Sudhir Bolla, Gerard J. Criner, Elise Messier, Nichole Reisdorph, Roger L. Powell, Muniswamy Madesh, Steven Kelsen, Nathaniel Xander, Kelly A. Correll, Robert J. Mason, Karim Bahmed

**Affiliations:** 1 Department of Thoracic Medicine and Surgery, Temple University, Philadelphia, PA 19140 USA; 2 Center for Inflammation, Translational and Clinical Lung Research, Temple University, Philadelphia, PA 19140 USA; 3 Department of Physiology, Temple University, Philadelphia, PA 19140 USA; 4 National Jewish Health, Denver, CO 80206 USA; 5 Medical Genetics and Molecular Biochemistry, Temple University, Philadelphia, PA 19140 USA

## Abstract

Emphysema is characterized by alveolar wall destruction induced mainly by cigarette smoke. Oxidative damage of DNA may contribute to the pathophysiology of this disease. We studied the impairment of the non-homologous end joining (NHEJ) repair pathway and DNA damage in alveolar type II (ATII) cells and emphysema development. We isolated primary ATII cells from control smokers, nonsmokers, and patients with emphysema to determine DNA damage and repair. We found higher reactive oxygen species generation and DNA damage in ATII cells obtained from individuals with this disease  in comparison with controls. We also observed low phosphorylation of H2AX, which activates DSBs repair signaling, in emphysema. Our results indicate the impairement  of NHEJ, as detected by low XLF expression. We also analyzed the role of DJ-1, which has a cytoprotective activity. We detected DJ-1 and  XLF interaction in ATII cells in emphysema, which suggests the impairment of their function. Moreover, we found that DJ-1 KO mice are more susceptible to DNA damage induced by cigarette smoke. Our results suggest that oxidative DNA damage and ineffective the DSBs repair via the impaired NHEJ may contribute to ATII cell death in emphysema.

## Introduction

Emphysema belongs to chronic obstructive pulmonary disease (COPD). Cigarette smoke is a main risk factor of this disease development^1^. However, the pathophysiology of emphysema is not fully understood^2^. It has been reported that an imbalance between proteases and anti-proteases may be involved in this disease development^3,4^. Moreover, emphysema is associated with an increased oxidative stress, which can cause DNA damage and alveolar type II (ATII) cell death^5^. ATII cells produce and secrete pulmonary surfactant, have a stem cell potential and restore the epithelium after damage^6–10^. Therefore, ATII cell injury can impair the function of anti-proteases and surfactant and contribute to emphysema development^11,12^. Furthermore, recent study showed an interplay between ATII cells and immune-related signaling events^13^. In addition, stimulation of ATII cells with TLR (Toll –like receptor) ligands leads to secretion of various chemokines and cytokines.

DNA double strand breaks (DSBs) can be caused by oxidative stress^14^. They activate DNA repair mechanisms to eliminate DNA damage. Cells cannot survive with even one unrepaired DSB, which indicates the importance of the functional DNA damage repair system^15^. Non-homologous end-joining (NHEJ) and homologous recombination (HR) are involved in DSBs repair^16^. HR is considered as the most accurate system^16,17^, however, NHEJ is likely playing the largest role in DSBs repair in humans, is also a faster and more efficient pathway than HR^18,19^. The NHEJ repair pathway includes Ku70, Ku86, DNA ligase IV, XRCC4 (X-ray repair cross-complementing protein 4), Artemis, XLF (XRCC4-like factor) and DNA-PKcs (DNA-dependent protein kinase, catalytic subunit). Induction of this system begins with the detection of DSBs by Ku proteins followed by 3 steps: synapsis by DNA-PKcs, end processing by nucleases such as Artemis and interaction of XLF with XRCC4, which leads to ligation of DNA ends by DNA ligase IV^20,21^.

DJ-1 is a conserved multifunctional protein, which has cytoprotective functions^22,23^. We have recently reported that the DJ-1 pathway modulates the activity of the antioxidant defense system in human primary ATII cells^24,25^. We showed that ATII cells isolated from heavy smokers and emphysema patients have high oxidative stress levels and apoptosis. This was correlated with the impairment of the DJ-1 protective activity. However, the role of DJ-1 in oxidative DNA damage in ATII cells in the pathogenesis of emphysema is not known.

Here we found, for the first time, that ATII cells isolated from patients with emphysema have higher ROS and DNA damage compared to control non-smokers or smokers. Therefore, we analyzed expression of proteins involved in both alternative and classical NHEJ pathways to determine the efficiency of DNA damage repair in ATII cells under high oxidative stress in this disease. We found downregulation of these proteins, which suggests that unrepaired DSBs lead to ATII cell death. Moreover, we identified DJ-1 as a novel interaction partner of XLF in ATII cells in emphysema. We hypothesize that this interaction may inhibit ROS scavenging function of DJ-1, leading to high oxidative DNA damage, the impairment of the NHEJ repair pathway, ATII cell death and this disease development.

## Results

### Increased ROS levels in ATII cells in emphysema patients

**Figure 1:**
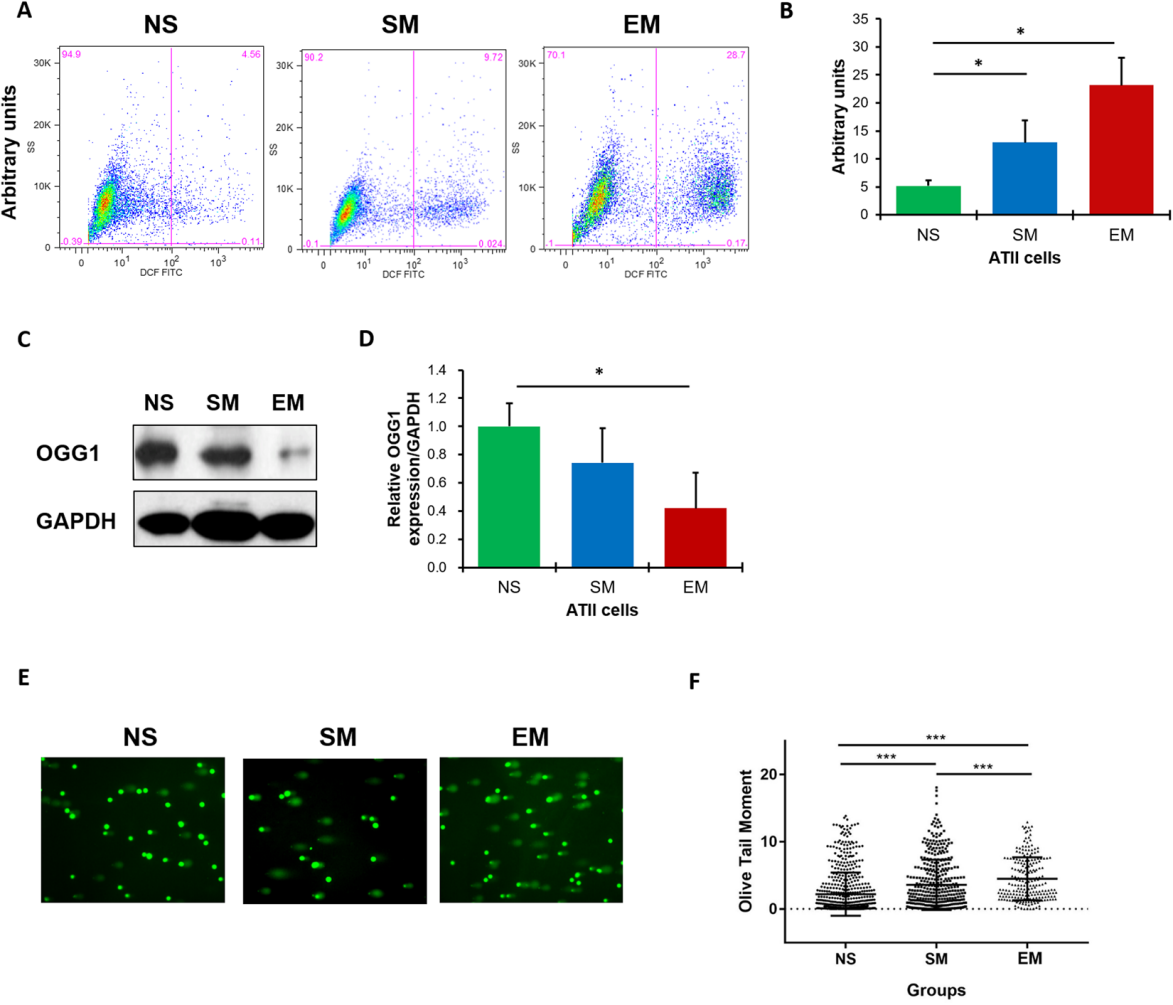
High ROS, DNA damage and the impairment of DNA damage repair in ATII cells in emphysema patients. ROS levels were analyzed in freshly isolated ATII cells from control non-smokers (NS), smokers (SM) and emphysema patients (EM) using DCF staining by flow cytometry. Representative images of flow cytometry profiles (**A**) and quantification (**B**) are shown. Representative Western blot images of OGG1 expression (**C**) and densitometric quantification of OGG1 expression normalized to GAPDH and non-smokers (**D**). Comet assay was used to detect DNA damage in freshly isolated ATII cells (**E**). Quantification of the Olive Tail Moment is also shown (**F**). Data are shown as mean values ± s.e.m. (N = 6 per group; *p < 0.05; ***p < 0.001, magnification 10 × 40).

Since decreased ATII cell proliferation and increased ATII cell death have been recently observed in COPD patients^26^, we wanted to determine ROS production in freshly isolated ATII cells from individuals with emphysema. Our results show significantly higher ROS levels in ATII cells in this disease in comparison with control non-smokers (Fig. 1A,B). This suggests that high ROS generation leads to ATII cell injury and may contribute to alveolar wall destruction.

### High DNA damage in ATII cells in patients with emphysema

The OGG1 (8-oxoguanine DNA glycosylase-1) is responsible for the excision of 8-oxoguanine (8-oxoG), a mutagenic byproduct that occurs as a result of cell exposure to ROS^27^. We found the lowest OGG1 levels in ATII cells in emphysema (Fig. 1C,D). This suggests decreased cell capacity to repair oxidative DNA damage caused by enhanced ROS production, which is in agreement with our results shown in Fig. 1A,B.

We also wanted to determine DNA damage in freshly isolated ATII cells. We found the characteristic “tails” of comets, which indicate DNA damage (Supplementary Fig. S1). The highest levels were observed in patients with emphysema as compared to control non-smokers or smokers (Fig. 1E,F). Our results indicate increased DNA damage in ATII cells in emphysema.

**Figure 2:**
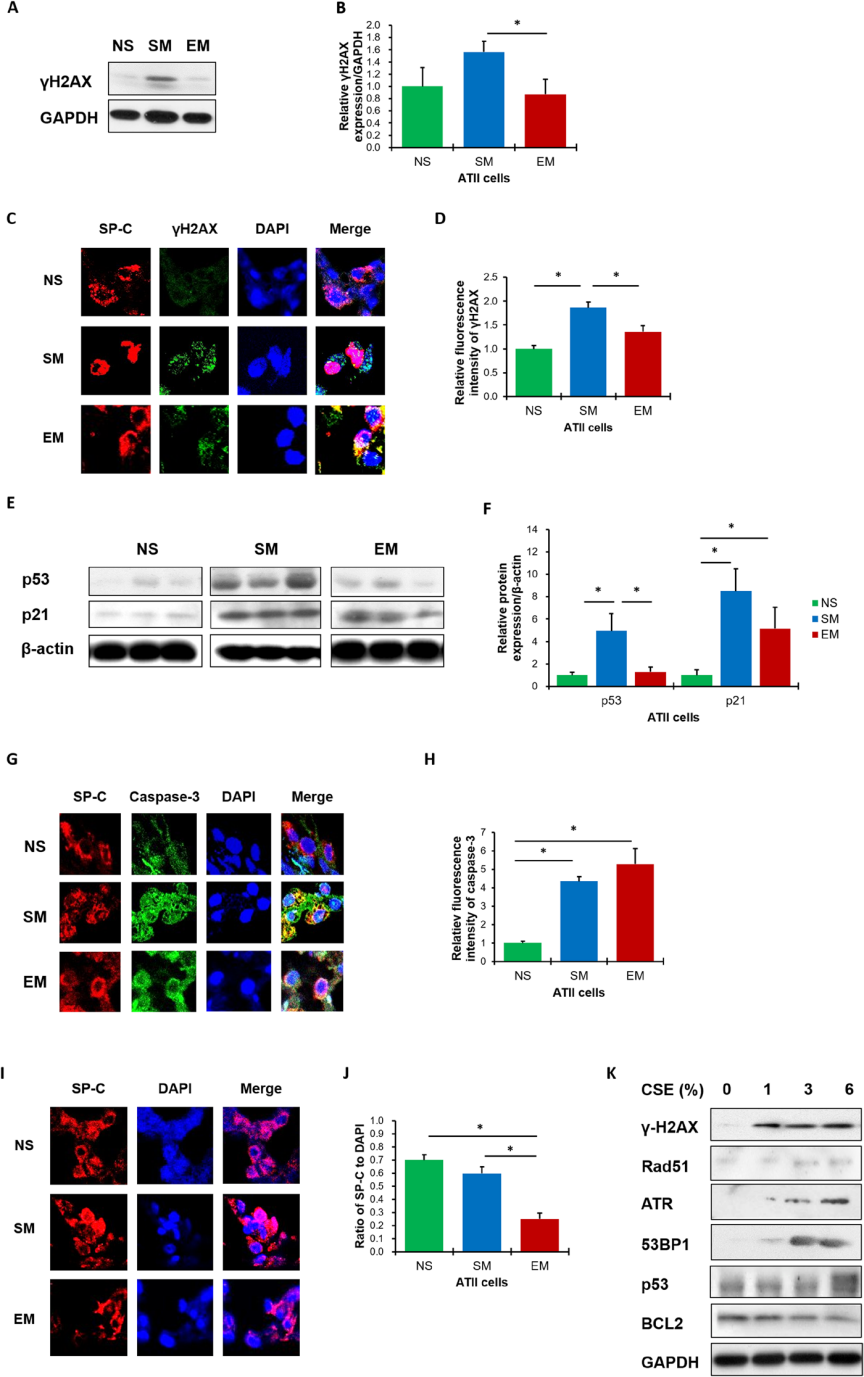
High DNA damage and the impairment of DNA damage repair in ATII cells in patients with emphysema. Freshly isolated ATII cells from non-smokers (NS), smokers (SM) and patients with emphysema (EM) were used to determine γH2AX expression by Western blotting (**A**). Densitometric quantification of γH2AX levels normalized to GAPDH and non-smokers (**B**). Human lung sections were co-stained for SP-C to identify ATII cells (red), γH2AX (green) and nuclei (DAPI, blue) by immunohistofluorescence (magnification 10 × 63) (**C**). The relative fluorescence intensity of γH2AX expression in ATII cells is also shown (**D**). Representative p53 and p21 expression from the same Western blotting (**E**) and densitometric analysis (**F**) in ATII cells obtained from non-smokers, smokers and emphysema patients. Apoptotic ATII cells were detected using SP-C (red), caspase 3 (green) and DAPI (blue) staining by immunohistofluorescence (**G**) and quantification is also shown (**H**). Decreased number of ATII cells using SP-C staining (red) in lung tissue sections from emphysema patients by immunohistofluorescence (DAPI, blue; magnification 10 × 63) (**I**) and quantification (**J**). Data are presented as means ±s.e.m (*p < 0.05). Cultured ATII cells isolated from control non-smokers were treated with CSE for 24 h (**K**). Lane 1 – control, lane 2–1% CSE, lane 3–3% CSE and lane 4–6% CSE. Cell lysates were subjected to Western blotting to determine protein expression (N = 3 per group).

Next we analyzed the phosphorylation of H2AX (*γ*H2AX), which activates DSBs repair signaling^28^. We found γH2AX phosphorylation in ATII cells obtained from smokers (Fig. 2A,B). Interestingly, ATII cells isolated from emphysema patients had lower γH2AX expression in comparison with smokers. This suggests the impairment of the DSBs repair signaling leading to decreased DNA damage repair in these cells. We also determined γH2AX levels in ATII cells by immunohistofluorescence. We detected higher γH2AX florescence intensity in ATII cells obtained from smokers in comparison with emphysema (Fig. 2C,D).

P53 is a transcription factor involved in apoptosis, cell cycle arrest and regulates expression of many genes involved in DNA damage repair pathways^29^. P21 binds to and inactivates cyclin/cyclin-dependent kinases (cdk) complexes, which cause cell cycle arrest^30^ and promote DNA damage repair. We found that ATII cells obtained from smokers have higher both p53 and p21 levels compared to non-smokers (Fig. 2E,F). We observed significantly decreased p53 expression in ATII cells in emphysema compared to smokers, which indicates the impairment of DNA damage repair pathway. Furthermore, ATII cells isolated from individuals with this disease had higher p21 levels than non-smokers. This suggests that persistent increase of p21 levels may induce a permanent cell cycle arrest leading to cell death. Moreover, we detected higher caspase 3 expression in ATII cells in individuals with emphysema in comparison with control smokers and non-smokers by immunohistofluorescence (Fig. 2G,H). Together, these data correlates with a lower number of ATII cells observed in individuals with this disease compared to controls (Fig. 2I,J), which indicates cell death.

### The impairment of DNA damage repair pathways in ATII cells in emphysema

We also wanted to check activation of DNA damage repair pathways *in vitro*. We cultured ATII cells isolated from control non-smokers followed by exposure to 1%, 3% or 6% cigarette smoke extract (CSE) for 24 h (Fig. 2K). We detected an increase in the phosphorylation of γH2AX and higher 53BP1 and ATR levels, which are involved in a classical NHEJ pathway. However, we did not observe changes in RAD51 expression, which is implicated in the HR pathway^31^. Moreover, we found decreased anti-apoptotic BCL2 levels with higher CSE concentrations. This correlated with increased DNA damage as detected by high p53 expression. Our data show that first, CSE induces DNA damage and second, a classical NHEJ may be involved in DNA damage repair.

**Figure 3:**
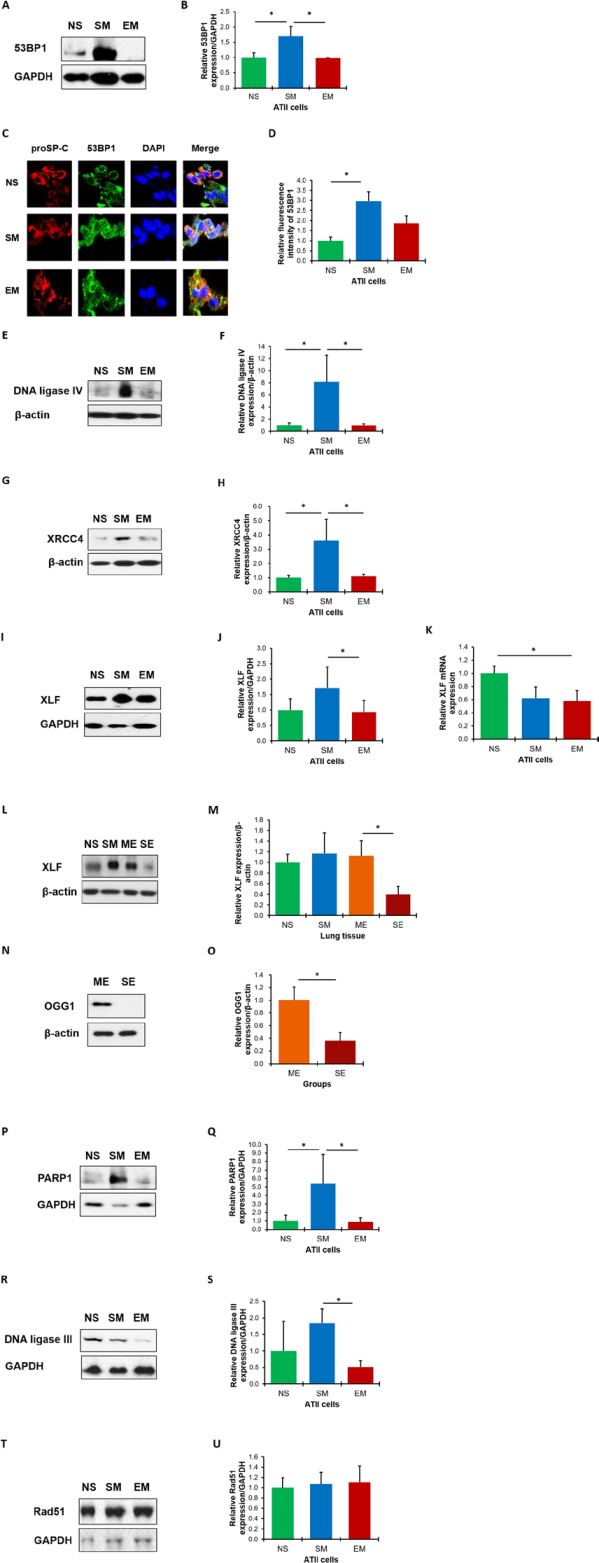
The impaired NHEJ DNA damage repair pathway in ATII cells and lung tissue in emphysema. 53BP1 expression in freshly isolated ATII cells from control non-smokers (NS), smokers (SM) and patients with emphysema (EM) was analyzed by Western blotting (**A**). Densitometric quantification is also shown (**B**). 53BP1 (green) expression is shown in ATII cells identified by proSP-C staining (red) in lung tissue sections (nuclei - DAPI, blue) by immunohistofluorescence (magnification 10 × 63) (**C**). Quantification of fluorescence intensity is also shown (**D**). DNA ligase IV (**E**,**F**) and XRCC4 (**G**,**H**) expression in freshly isolated ATII cells was determined by Western blotting and densitometric quantification. XLF levels in ATII cells were determined by Western blotting (**I**), densitometric quantification (**J**) and RT-PCR (**K**). XLF levels were analyzed in lung tissue obtained from control non-smokers and smokers, and areas with mild (ME) and severe emphysema (SE) from the same patient by Western blotting (**L**,**M**). Expression of OGG1 (**N**,**O**) was analyzed in lung tissue obtained from areas with mild and severe emphysema from the same patient by Western blotting and quantification. Representative Western blotting images and densitometric quantification are shown for PARP1 (**P**,**Q**), DNA ligase III (**R**,**S**) and RAD51 expression (**T**,**U**) in freshly isolated ATII cells. Data are shown as mean values ± s.e.m. (N = 7 per group; *p < 0.05).

Next, we wanted to determine 53BP1, DNA ligase IV, XRCC4 and XLF expression, which form a complex to ligate the broken ends during the NHEJ process, in freshly isolated ATII cells from non-smokers, smokers and patients with emphysema. Our results indicate significantly higher 53BP1 levels in ATII cells in smokers compared to non-smokers as detected by Western blotting (Fig. 3A,B) and immunohistofluorescence (Fig. 3C,D). We found a similar correlation for DNA ligase IV (Fig. 3E,F) and XRCC4 expression (Fig. 3G,H) and their low expression in emphysema. We also observed decreased XLF protein (Fig. 3I,J) and mRNA levels (Fig. 3K) in ATII cells in individuals with this disease. These results indicate that ATII cells in emphysema have an impaired classical NHEJ pathway, which causes ineffective repair of DNA damage leading to cell death.

We also used lung tissue obtained from areas with mild and severe emphysema from the same patients after lung transplant to determine the correlation between disease severity and NHEJ (Supplementary Fig. S2). Interestingly, we found decreased XLF expression in severe compared to mild areas of emphysema (Fig. 3L,M). This indicates the correlation between the impairment of the NHEJ pathway and this disease progression. Moreover, OGG1 levels were also reduced in severe compared to mild emphysema (Fig. 3N,O).

It has been reported that an alternative NHEJ may be also involved in DSBs repair^32^. Therefore, we analyzed expression of PARP1 (Fig. 3P,Q) and DNA ligase III (Fig. 3R,S). We found their significantly decreased levels in ATII cells in emphysema compared to smokers. Our results indicate that the impairment of both classical and alternative NHEJ pathways contribute to low DSBs repair in this disease.

DSBs can also be repaired by HR pathway therefore, we determined RAD51 expression. However, we did not detect significant differences in RAD51 levels in ATII cells isolated from non-smokers, smokers and patients with emphysema (Fig. 3T,U). Our results strongly suggest that the inactivation of the NHEJ repair pathway contributes to DNA damage, low DNA damage repair and ATII cell death in this disease (Supplementary Fig. S3).

### The cytoprotective role of DJ-1

**Figure 4:**
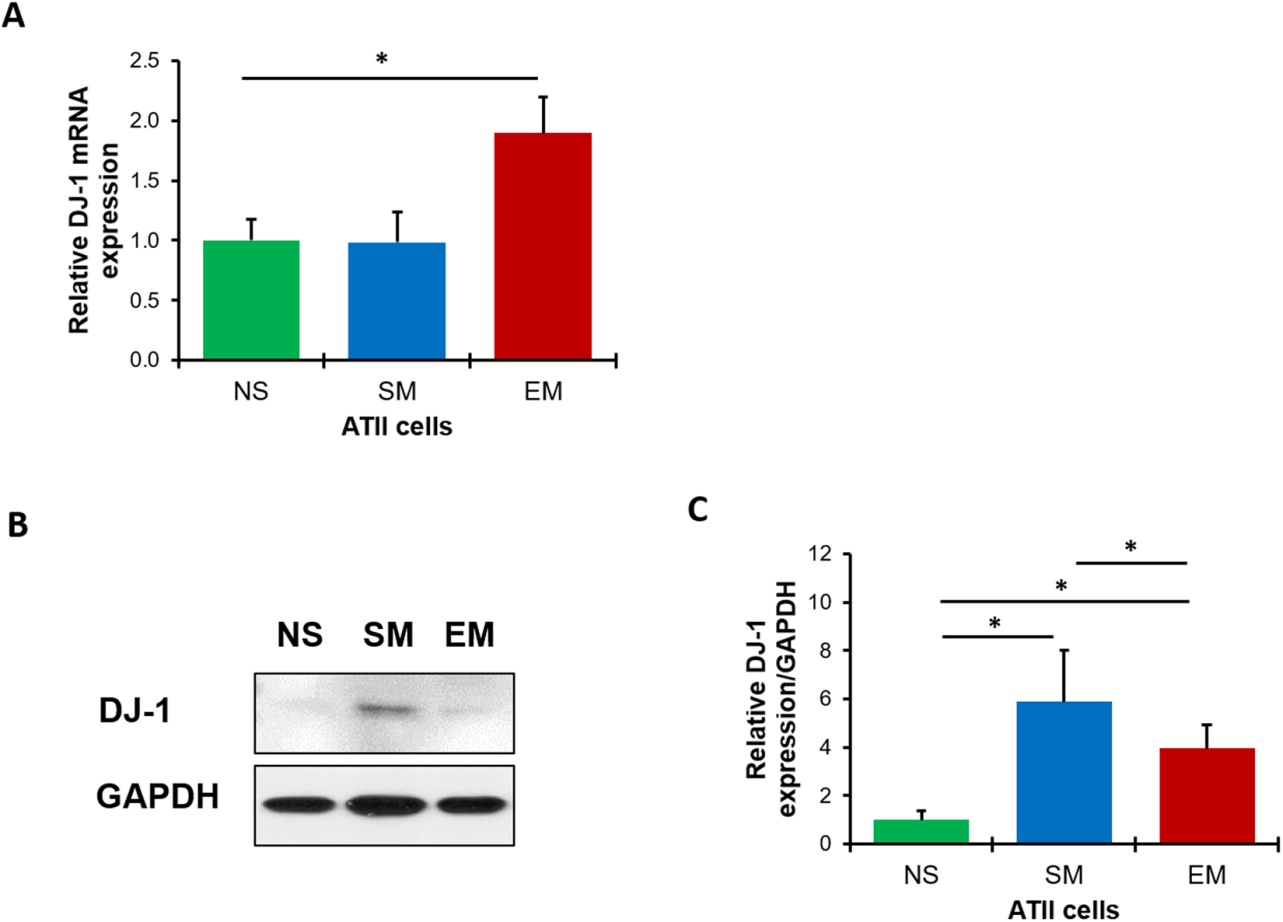
DJ-1 expression in human ATII cells. DJ-1 expression was analyzed by RT-PCR (**A**), Western blotting (**B**) and densitometric analysis (**C**) in freshly isolated ATII cells from non-smokers (NS), smokers (SM) and patients with emphysema (EM). Data are shown as mean values ± s.e.m. (N = 6 per group; *p < 0.05).

DJ-1 is a multifunctional protein with antioxidant activity; however, its cytoprotective function against DNA damage is not well known. We found higher DJ-1 mRNA expression in emphysema compared to controls as detected by RT-PCR (Fig. 4A). However, we observed decreased DJ-1 protein expression in ATII cells isolated from individuals with emphysema in comparison with control smokers (Fig. 4B,C). This indicates the correlation between reduced cytoprotective function of DJ-1 and an increased DNA damage.

### XLF interaction with DJ-1

**Figure 5:**
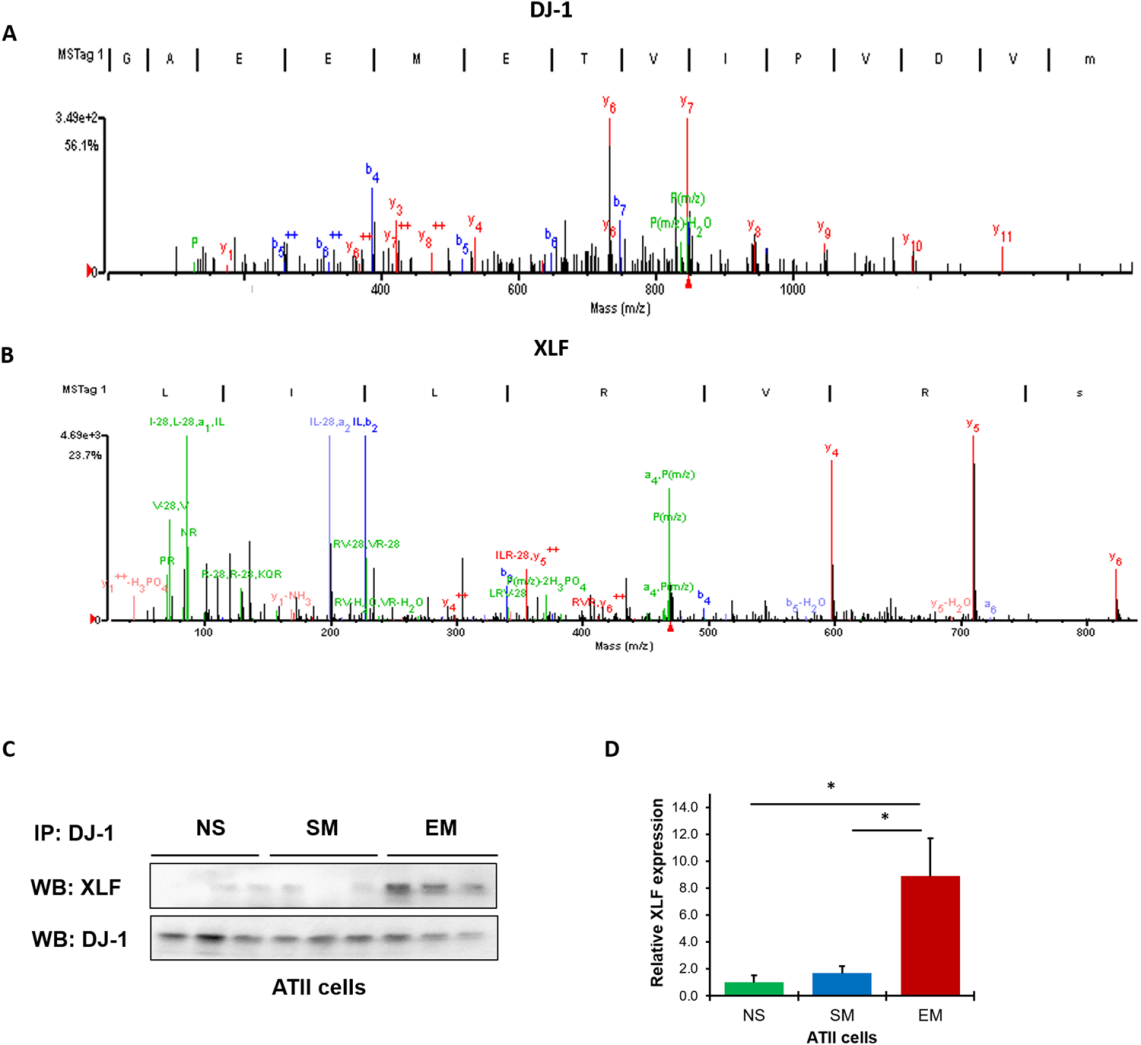
DJ-1 and XLF interaction in freshly isolated ATII cells from patients with emphysema identified by mass spectrometry analysis. DJ-1 was co-immunoprecipitated in ATII cells obtained from non-smokers (NS), smokers (SM) and patients with emphysema (EM) followed by mass spectrometry analysis. The presence of DJ-1 (**A**) and its interaction with XLF (**B**) is shown in emphysema (N = 3 per group). Results were validated by co-immunoprecipitation of DJ-1 followed by Western blotting analysis of XLF expression in ATII cells obtained from non-smokers, smokers and patients with emphysema (**C**) and densitometric quantification (N = 6 per group; *P < 0.05) (**D**). Data is shown as means ± s.e.m.

To further study the role of DJ-1 in ATII cells in emphysema, we used a proteomic approach. We discovered XLF as one of the most abundant DJ-1 binding partners in ATII cells obtained from individuals with emphysema by mass spectrometry analysis (Fig. 5A,B). Interestingly, we did not detect this interaction in control non-smokers (data not shown). We validated our obtained results by immunoprecipitation of DJ-1 in ATII cells followed by Western blotting using XLF antibody. We found a similar correlation and observed significantly higher DJ-1 and XLF interaction in ATII cells in emphysema compared to control non-smokers and smokers (Fig. 5C,D). Our results suggest that this interaction may inhibit XLF activity and/or stability and block XLF access to DSBs leading to decreased DNA damage repair. Our results indicate first, the participation of DJ-1 in NHEJ and second, the impairment of cytoprotective function of DJ-1 in emphysema.

### The role of XLF in DJ-1 KO mice exposed to cigarette smoke

**Figure 6:**
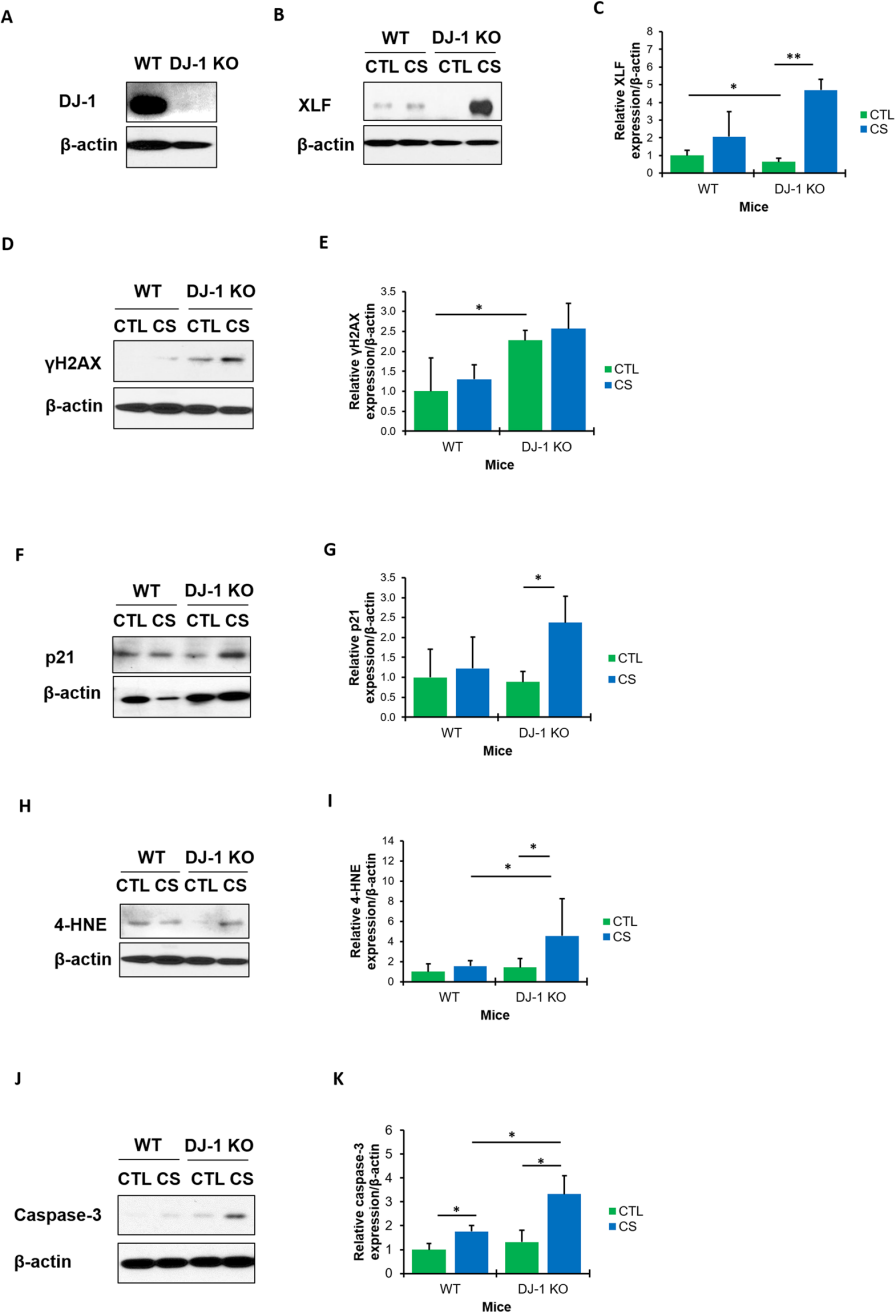
XLF and DJ-1 expression in lung tissue obtained from mice exposed to cigarette smoke *in vivo*. Lung tissue was obtained from wild-type (WT) and DJ-1 KO mice exposed to 150 mg/m^3^ TSP as described in Material and Method section (CTL – control; CS-cigarette smoke). The absence of DJ-1 expression in DJ-1 KO mice was confirmed by Western blotting (**A**). Representative Western blot images of expression and densitometric analysis of XLF (**B**,**C**), γH2AX (**D**,**E**), p21 (**F**,**G**), 4-HNE (**H**,**I**) and caspase 3 (**J**,**K**) are shown. Densitometric quantification of protein expression was normalized to β-actin (N = 3 per group). *P < 0.05; **P < 0.001. Data is shown as means ± s.e.m.

We wanted to further determine DJ-1 and XLF functions under oxidative stress *in vivo*. We used lung tissue obtained from wild-type and DJ-1 KO mice (Fig. 6A) exposed to cigarette smoke. First, we observed significantly lower XLF levels in control DJ-1 KO mice compared to wild-type mice by Western blotting (Fig. 6B,C). Second, exposure to cigarette smoke significantly increased XLF levels in DJ-1 KO mice in comparison with control.

We also found significantly increased γH2AX expression in control DJ-1 KO mice compared to wild-type mice (Fig. 6D,E). Moreover, DJ-1 KO mice exposed to cigarette smoke showed higher γH2AX levels than wild-type mice, although there was some variation. We also found increased p21 (Fig. 6F,G), 4-HNE expression (Fig. 6H,I) and caspase 3 levels (Fig. 6J,K) in DJ-1 KO mice exposed to cigarette smoke, which suggests high DNA damage, oxidative stress and apoptosis, respectively. Our obtained results suggest that DJ-1 KO mice are more susceptible to oxidative DNA damage compared to wild-type mice and also indicate for the first time the functional interplay between XLF and DJ-1 in the lung.

## Discussion

Emphysema involves irreversible damage of the alveolar wall^33,34^. The impaired ATII cell proliferation, injury and death caused by cigarette smoke are critical factors, which contribute to this disease development. To our knowledge, there are no studies on DNA damage and repair using isolated ATII cells obtained from patients with emphysema.

The level of intracellular ROS is regulated by the balance between pro-oxidant and antioxidant systems^35^. High oxidative stress can cause DNA damage. Accumulation of unrepaired DNA damage induced by ROS^36,37^ may contribute to emphysema pathogenesis.

Our results show that ATII cells isolated from emphysema patients have higher ROS levels and DNA damage as detected by DCF staining and comet assay, respectively. We also found decreased expression of proteins involved in DNA damage repair (such as 53BP1, DNA ligase IV, XRCC4, XLF and γH2AX) in ATII cells obtained from individuals with this disease. Lower expression of these enzymes with simultaneously higher oxidative stress, DNA damage and apoptosis indicates an impaired DNA damage repair. DSBs are the most dangerous type of DNA damage because they are sufficient to induce cell death^38,39^. We have shown that p21 is upregulated and p53 is downregulated in ATII cells in individuals with emphysema. High expression of p21 may be caused by very high oxidative stress and suggests deficient cell cycle progression and longer or permanent arrest to repair oxidative DNA damage. Moreover, we did not detect activation of DNA damage repair in ATII cells in emphysema as shown by low levels of OGG1, γH2AX and XLF. Our data is in agreement with other reports showing that p21 can trigger cell death in p53-independent manner^40^.

Efficient repair of DSBs relies on two major pathways: HR and NHEJ. HR is using the corresponding undamaged DNA template present on the homologous chromosomes. On the other hand, NHEJ is involved in a direct ligation of the two broken ends^41^. The impairment of either NHEJ or HR pathways leads to cell death. HR is an error-free process, which operates in proliferating cells. During the G1 phase, HR is inactivated and NHEJ is a dominant system. In the S and G2 phases, when sister chromatids of chromosomes are available, NHEJ and HR compete to repair DNA damage. This indicates that NHEJ is an error prone mechanism that can function in both proliferating and differentiated cells. The recombinase RAD51 belongs to HR pathway and facilitates homology search and DNA strand invasion. However, we did not detect differences in RAD51 expression in ATII cells isolated from emphysema patients in comparison with controls. Considering the fact that we found high DSBs in ATII cells in individuals with this disease, our results suggest that HR pathway is ineffective in DNA damage repair in these cells.

We detected high ROS levels in emphysema, which indicate oxidative DNA damage. DSBs induce phosphorylation of γH2AX leading to recruitment of proteins involved in DNA damage repair^42–45^. We found significantly lower γH2AX levels in ATII cells isolated from emphysema patients compared to control smokers. It has been reported that decreased γH2AX expression indicates inefficient DSBs repair^44^. Moreover, we observed lower XLF, 53BP1, DNA ligase III, DNA ligase IV and PARP1 expression in ATII cells in emphysema in comparison with control smokers. Our results suggest that high oxidative stress in ATII cells in this disease contributes to the impaired NHEJ repair pathway. Furthermore, we found a decreased number of ATII cells in lung tissue in emphysema by immunocytofluorescence, which is in agreement with recent studies showing lower ATII cell proliferation in COPD^26^. Together, our results suggest that DNA damage induced by high ROS generation^46,47^ leads to deficient NHEJ and ATII cell death, which may contribute to emphysema progression.

NHEJ and HR pathways are also involved in the maintenance of DNA stability. Therefore, defects in one of these systems can contribute to carcinogenesis^48^. Of note, patients with COPD have an increased risk of lung cancer development^49–51^, which is one of the most common causes of mortality among these individuals^52^.

DJ-1 has cytoprotective and ROS scavenging activity^22^. We have previously shown that DJ-1 is involved in the induction of the antioxidant defense system in human primary ATII cells in response to oxidative stress caused by cigarette smoke^24,25^. Here, we found that DJ-1 is downregulated in ATII cells isolated from individuals with emphysema. Moreover, it interacts with XLF in ATII cells obtained from these patients. This interaction suggests that DJ-1 may inhibit XLF activity to efficiently repair DNA damage. This correlates with high ROS levels and DNA damage detected in this disease. We also found lower XLF expression in areas with severe compared to mild emphysema, which suggests that impaired DNA damage repair contributes to this disease progression (Supplementary Fig. S4).

We also used lung tissue obtained from wild-type and DJ-1 KO mice exposed to cigarette smoke. First, we found high oxidative stress as detected by increased 4-HNE levels in DJ-1 KO mice. Second, we observed significantly lower expression of XLF in control DJ-1 KO mice compared to wild-type mice. Third, we detected that exposure to cigarette smoke increased XLF levels in DJ-1 KO mice. Our results suggest that cigarette smoke-induced oxidative stress leads to high DNA damage in the absence of DJ-1. We observed that this exposure activates XLF expression and DSBs repair, which indicates that XLF may serve as a compensatory mechanism to accelerate and promote DNA damage repair in DJ-1 KO mice.

Our results show higher γH2AX levels in control DJ-1 KO mice in comparison with wild type mice. Moreover, exposure to cigarette smoke increased p21 levels in DJ-1 KO mice. However, we did not detect significant changes in wild-type mice. The discrepancy between p21, XLF and γH2AX levels in mice and human can be explained by the duration of exposure to cigarette smoke. Mice were exposed for 2 h per day for 3 weeks. However, lung tissue was obtained from smoker organ donors who smoked 5–20 cigarettes per day for at least 3 years. Together, our results suggests that the absence of DJ-1 increases oxidative stress, DNA damage and activates XLF-dependent DNA damage repair pathway.

## Methods

### ATII cell isolation and culture

Human lungs, obtained from deidentified control non-smoker and smoker organ donors were donated for medical research by the Gift of Life Donor Program. We selected donors with lung function with a PaO_2_/FIO_2_ ratio of >250, a clinical history and x-ray that did not indicate infection, and limited time on a ventilator. Non-smokers were individuals who never smoked and smokers smoked 5–20 cigarettes per day for at least 3 years. Lung tissue from patients with emphysema (GOLD IV) was obtained from lung transplantation performed at Temple University, Philadelphia, PA (N = 4–6 per group, age 49–62 years old, females and males). ATII cells were isolated as we previously described^24,53–55^. We cultured ATII cells as we reported^56^. The purity of ATII cells was ~95% after adherence in culture as we determined by staining for cytokeratin CAM 5.2^57^. All experiments were performed in accordance with relevant guidelines and regulations. The informed consent was obtained from all patients. The study was performed in accordance with the Declaration of Helsinki and was approved by the Institutional Review Boards at Partners Healthcare and the Committee for the Protection of Human Subjects at Temple University.

### Chest CT scans

Patients underwent volumetric CT scans of the chest at full inspiration (standard dose = 200 mAs) and at end-tidal expiration (low dose = 50 mAs). Detailed CT protocols have been previously published^58^. Performed CT scans were subjected to a standard quality control procedure. We used 3D SLICER software for computerized image analysis^59^. Emphysema was quantified by the percent of the lung voxels on inspiratory CT scan with attenuation <−950 HU (Insp−950)^60^. It was considered absent in subjects with values for Insp−950 < 4% in smokers, to account for the fact that the increased lung density in smokers results in a decrease in emphysema index^61^. We defined severe emphysema by Insp−950 > 14% in smokers. Lung tissue cores were obtained from areas with mild and severe emphysema after transplantation from the same patient (N = 7) as previously described^62^.

### Flow cytometry analysis

ROS levels in ATII cells were measured using DCF-DA (2.7-dichlorofluorescein diacetate, Sigma-Aldrich, St. Louis, MO) probe. DCF-DA is a stable non-fluorescent, cell permeable compound, which enters the cells and is converted to a highly fluorescent DCF compound by its reaction with ROS. We used 1 × 10^5^ ATII cells isolated from non-smokers, smokers and emphysema patients followed by incubation with 10 µM DCF-DA for 30 min at 37 °C. Cells were washed with PBS and re-suspended in a total volume of 500 µl. Cells were analyzed by LSR-II flow cytometer (BD Biosciences, San Jose, CA) and FlowJo software (Tree Star, Inc).

### RT-PCR

RNA was isolated from ATII cells and lung tissue obtained from patients with emphysema and controls using Quick-RNA MiniPrep (Zymo Research, Irvine, CA) according to the manufacturer’s instructions. Briefly, 1 μg of RNA was added to oligo d(T)20 primers and dNTP mix for 5 min at 65 °C. Reverse transcription reaction was performed at 55 °C for 10 min followed by 80 °C for 10 min. RT-PCR was used to analyze mRNA expression of human DJ-1 and XLF genes. Primers were retrieved from PrimerBank (http://pgamgh.harvard.edu/primerbank/) and ordered from Invitrogen (Waltham, MA). We used XLF forward 5′ GGCCAAGGTTTTTATCACCAAGC and reverse 5′ TGGGCGAAGGAGATTATCCAAAT 3′; DJ-1 forward 5′ GTAGCCGTGATGTGGTCATTT 3′ and reverse 5′ CTGTGCGCCCAGATTACCT 3′; and GAPDH forward 5′ GGAGCGAGATCCCTCCAAAAT 3′ and reverse 5′ GGCTGTTGTCATACTTCTCATGG 3′. We applied SuperScript IV First-Strand Synthesis System (Thermo Fisher Scientific, Waltham, MA) for the cDNA synthesis according to the manufacturer’s recommendations. RT-PCR was performed by using the SYBR Green Master Mix kit (Thermo Fisher Scientific, Waltham, MA) and GAPDH was applied as an internal control. Thermal cycle conditions were: denaturation at 95 °C for 10 min followed by 45 cycles of 95 °C for 15 s, 58 °C for 60 s and 68 °C for 20 s. Obtained results were analyzed using the ΔΔCt method. The expression levels of DJ-1 and XLF were measured in triplicate from at least three independent experiments.

### Western Blotting and Immunoprecipitation

ATII cells or lung tissue lysates were prepared using PE-LB lysis buffer with protease and phosphatase inhibitor cocktail (all from Gold Biotechnology, Olivette, MO). Western blotting was performed as we previously described^24^. Briefly, lysates were separated by 8–16% polyacrylamide gradient gels (Thermo Fisher Scientific, Waltham, MA) and proteins were transferred using nitrocellulose membrane. We used 5% bovine serum albumin for blocking membranes followed by incubation with primary antibodies overnight at 4 °C. Horseradish peroxidase conjugated with secondary antibody was used on the following day. Protein expression was detected by Luminata Forte Western HRP Substrate (Millipore, Billerica MA). We obtained GAPDH from Abcam (Cambridge, MA) and β-actin from Sigma-Aldrich (St. Louis, MO). Anti-53BP1, γH2AX, DNA ligase III, DNA ligase IV, OGG1, XLF, XRCC4, RAD51, p53, p21, ATR, PARP1 and DJ-1 antibodies were purchased from Santa Cruz Biotechnology (Santa Cruz, CA) and used for Western blotting as we previously described^24^. For immunoprecipitation, we used Protein A/G Plus Agarose beads (Santa Cruz Biotechnology, CA) to collect the immunoprecipitates. Densitometric analysis of the obtained bands was quantified using ImageJ and NIH Image 1.62 software (Bethesda, MD, USA). For a positive control, ATII cells were irradiated with 100 J of 254 nm UV light/m^2^ (Spectrolinker XL-1500) for 5 min. ATII cells were harvested after 3 h post incubation and prepared for Western blotting as described above.

### Cigarette smoke extract

The cigarette smoke extract was prepared using one 3R4F reference cigarette (Kentucky Tobacco Research & Development Center, Lexington, KY) as we previously described^57^. Obtained extract was immediately used for cell treatment for 24 h.

### Immunohistofluorescence

Human lung tissue obtained from non-smokers (NS), smokers (SM), and emphysema patients (EM) was fixed in 4% paraformaledyde and embedded in paraffin. Sections were deparaffinized and hydrated followed by antigen retrieval by boiling in 0.01 M citrate buffer for 10 min and blocking with 3% normal donkey serum (Sigma-Aldrich, St. Louis, MO) for 20 min. Lung tissue sections were incubated with surfactant protein C (SP-C; Santa Cruz Biotechnology, CA), proSP-C (Millipore, Billerica MA), γH2AX, caspase 3 or 53BP1 overnight followed by adding corresponding fluorochrome-labeled secondary antibodies: Alexa Fluor 594 IgG and Alexa Fluor 488 IgG (Invitrogen, Waltham, MA) for 1 h. Fluoroshield Mounting Medium containing DAPI (Abcam, Cambridge, MA) was used to detect nuclei. Sections were analyzed using a confocal laser-scanning microscope (Zeiss) and the fluorescence intensity^63^ was analyzed and quantified by ImageJ (NIH, Bethesda, MD). We performed parallel immunohistofluorescence staining for all slides. We selected ATII cells to analyze fluorescence intensity of protein of interests by applying ImageJ and NIH Image 1.62 software. Identical image acquisition settings and exposure times were used. We calculated the corrected total cell fluorescence (CTCF) using the following formula: CTCF = Integrated Density − (Area of selected cell X Mean fluorescence of background readings).

### Mouse exposure to cigarette smoke

The wild-type C57BL/6 and DJ-1 knockout (KO) mice were purchased from the Jackson Laboratory (Bar Harbor, ME). All mice were fed ad libitum and housed in an Institutional Animal Care and Use Committee (IACUC)-accredited facility in individually ventilated cages. Mice were age- and sex-matched and randomly assigned to control or treatment groups (n = 4). We used 6-week-old mice with male: female ratio = 1:1 for all experiments. Animals were exposed to cigarette smoke generated from 3 R4F reference cigarettes (Kentucky Tobacco Research & Development Center, Lexington, KY) for 2 h per day for 3 weeks using a Teague TE-10 smoking system (Teague Enterprises, Woodland, CA). The total suspended particulate (TSP) was 150 mg/m^3^, and CO concentration was less than 300 ppm. All experimental procedures were approved by the IACUC at Temple University. Animal care, handling and experimental procedures were carried out in accordance with a protocol approved by the IACUC of Temple University.

### Comet Assay

Measurement of DNA damage in the individual nuclei of freshly isolated ATII cells from control non-smokers, smokers and patients with emphysema was performed using the OxiSelect Comet Assay (Cell Biolabs, Inc., San Diego, CA) to detect DNA damage (single-stranded and double-stranded DNA breaks and AP sites). We followed the manufacturer’s instructions. Briefly, an aliquot of 1 × 10^5^ cells was mixed with LMP agarose followed by incubation with lysis and alkaline buffers to relax and denature DNA. The slides were placed in an electrophoresis unit in a horizontal chamber and electrophoresis was conducted for 30 min at 0.73 V/cm to separate an intact and fragmented DNA. After that, slides were dried, stained with DAPI (Abcam, Cambridge, MA) and covered with cover slips. Pictures were taken using fluorescence microscopy (Zeiss Axioskop 2, Carl Zeiss, Germany) and analyzed by OpenComet software^64^. DNA damage was quantified by measuring the displacement between the genetic material of the nucleus (‘comet head’) and the resulting ‘tail’. We calculated Olive Tail Moment in at least 300 nuclei from randomly selected images using the following formula:

$${\rm{Olive}}\,{\rm{Tail}}\,{\rm{Moment}}={\rm{Tail}}\,{\rm{DNA}} \% \,\times \,{\rm{Tail}}\,{\rm{Moment}}\,{\rm{Length}}.$$

$${\rm{Tail}}\,{\rm{DNA}} \% =100\,\times \,{\rm{Tail}}\,{\rm{DNA}}\,{\rm{Intensity}}/{\rm{Cell}}\,{\rm{DNA}}\,{\rm{Intensity}}.$$

Tail Moment Length was measured from the center of the comet head to the center of its tail. For a positive control, ATII cells were isolated from non-smokers followed by treatment with 100 µM H_2_O_2_ (Sigma-Aldrich, St. Louis, MO) for 1 h.

### Mass spectrometry analysis

DJ-1 antibody was used to perform immunoprecipitation followed by Western blotting. We applied a standard protein identification strategy in freshly isolated ATII cells from control non-smokers and smokers as well as patients with emphysema using mass spectrometry analysis^65^.

### Statistics

We used one-way ANOVA to determine statistically significant differences (*p* < 0.05). Data is shown as means ± s.e.m. from at least three independent experiments.

## Supplementary information

Supplementary information

## Data Availability

All data generated or analyzed during this study are included in this article and its Supplementary Information files. Cropped images of Western blots are shown in all figures. Full-length images of Western blots are included in Supplementary Material.
