# Supplementary material for: Impaired non-homologous end joining in human primary alveolar type II cells in emphysema
Source: Sci Rep. 2019 Jan 29;9:920. doi: 10.1038/s41598-018-37000-z (PMC6351635; doi:10.1038/s41598-018-37000-z)
Supplement: Supplementary file 1 — Supplementary information [file 41598_2018_37000_MOESM1_ESM.pdf]

Supplementary Information

Impaired non-homologous end joining in human primary alveolar type II cells in emphysema

Beata Kosmider[1,2,3,*], Chih-Ru Lin[1,2], Liudmila Vlasenko[1,2], Nathaniel Marchetti[1,2], Sudhir Bolla[1], Gerard J. Criner[1,2], Elise Messier[4], Nichole Reisdorph[4], Roger L. Powell[4], Muniswamy Madesh[5], Steven Kelsen[1,2], Nathaniel Xander[2], Kelly A. Correll[4], Robert J. Mason[4], Karim Bahmed[1,2,*]

[1]Department of Thoracic Medicine and Surgery, Temple University, Philadelphia, PA 19140
[2]Center for Inflammation, Translational and Clinical Lung Research, Temple University, Philadelphia, PA 19140
[3]Department of Physiology, Temple University, Philadelphia, PA 19140
[4] National Jewish Health, Denver, CO, 80206
[5]Medical Genetics and Molecular Biochemistry, Temple University, Philadelphia, PA 19140

* Correspondence:

Beata Kosmider, Ph.D. or Karim Bahmed, PhD

Department of Thoracic Medicine and Surgery

Center for Inflammation, Translational and Clinical Lung Research

Temple University

3500 N. Broad Street

Philadelphia, PA 19140

E-mail: beata.kosmider@temple.edu or karim.bahmed@temple.edu

Supplementary Methods

ATII cell isolation and culture

ATII cells were isolated using elastase (Roche Diagnostics, Indianapolis, IN) instillation. The lung was minced and the cells were filtrated. The cell suspension was purified by centrifugation using a density gradient made of Optiprep (Accurate Chemical Scientific Corp., Westbury, NY) followed by negative selection with CD14-coated magnetic beads (Dynal Biotech ASA, Oslo, Norway). We used IgG-coated (Sigma-Aldrich, St. Louis, MO) dishes for this binding. The isolated ATII cells were plated with DMEM (GE Healthcare, Bensalem, PA) supplemented with 2 mM glutamine, 100 μg/ml streptomycin, 100 U/ml penicillin, 2.5 μg/ml amphotericin B and 10 μg/ml gentamicin (all from Thermo Scientific, Waltham, MA) on millicell inserts (Millipore Corp., Bedford, MA) coated with a mixture of 20% Matrigel Matrix (Corning, Tewksbury, MA) and 80% rat-tail collagen. ATII cell differentiated state was maintained by plating for 2 d with DMEM with 10% fetal bovine serum (FBS, GE Healthcare, Bensalem, PA) and additives as mentioned above. ATII cells were then cultured for 2 d with 1% charcoal-stripped FBS along with 10 ng/ml keratinocyte growth factor (KGF, R&D Systems Inc., Minneapolis, MN), and for additional 2 d with 1% charcoal-stripped FBS along with 10 ng/ml KGF, 0.1 mM isobutylmethylxanthine, 0.1 mM 8-bromo-cyclic AMP, and 10 nM dexamethasone (all from Sigma-Aldrich, St. Louis, MO).

Chest CT scans

Lungs were removed from the thorax, inflated with air and frozen using liquid nitrogen vapor followed by cutting into 2-cm thick slices in the same plane as the CT scan. Lung tissue cores

were collected from areas with mild and severe emphysema using a sharpened steel cylinder diameter of 1 cm. They were processed for Western blotting as descried below. Subjects provided written informed consent prior to surgery for use of these specimens and the relevant clinical and radiological data required for research.

Mass spectrometry analysis

Protein bands were excised from the gel using a sterile scalpel blade and cut into approximately 1 mm pieces. Excess liquid was removed and gel pieces were destained using 100 µl of 50% acetonitrile (ACN) and 25 mM ammonium bicarbonate (ABC). Gel pieces were shaken at room temperature for 10 min and the liquid removed. Following 3 destaining steps, 100 µl ACN was added, samples were incubated for 3-5 min, and all excess liquid was removed. Samples were dried in a biosafety cabinet for 5-10 min. 100 µl of fresh reductant (1.5 mg dithiothreitol (DTT) in 1 ml 25 mM ABC) was added and samples were incubated for 30 min at room temperature. The supernatant was removed and 100 µl fresh alkylating agent (10 mg iodoacetamide (IAA) in 1 ml 25 mM ABC) was added and samples were incubated for 30 min at room temperature in the dark. After removing the liquid, samples were washed with 100 µl 50 mM ABC and then with 100 µl 50% ACN in 25 mM ABC. 100 µl neat ACN was added until gel pieces were dehydrated and then removed and samples were dried for 5-10 min. To digest the proteins, 15 µl of trypsin (25 ng/µl) was added to rehydrate the gel pieces and remained at room temperature for 10-15 min. Excess liquid was removed and 25 mM ABC was added to cover the gel pieces. Samples were digested at 37°C overnight in an air incubator. To extract peptides, 1 µl of 10% formic acid was added and the pieces were shaken at room temperature for 10 min. The liquid containing peptides was transferred to a new microfuge tube and placed on ice. 60% ACN/0.1% formic acid was added to cover the gel pieces and the tubes were shaken for an additional 10 min at room

temperature. Liquid was combined with the previous liquid sample containing peptides. 100% ACN was then added to dehydrate the gel pieces; remaining liquid was added to the previous tube and samples were then dried in a speedvac at 55°C. Dried samples containing tryptic peptides were re-suspended in 10 µl of 3% ACN/0.1% formic acid, transferred to auto sampler vials and analyzed by mass spectrometry. Peptides were chromatographically resolved on-line using a C18 column and 1,200 series high performance liquid chromatography (HPLC, Agilent Technologies) and analyzed using a 6520 LCMS QTOF mass spectrometer (Agilent Technologies, Palo Alto, CA) with an HPLC-chip interface (Agilent Technologies). Buffer A of the nanopump was comprised of HPLC grade water and 0.1% formic acid, buffer B was 90% acetonitrile, 10% HPLC water, and 0.1% formic acid. The loading pump utilized 3% acetonitrile, 97% HPLC grade water and 0.1% formic acid. An HPLC chip (Agilent G4240-62002) was used which consisted of a 40 nL enrichment column and a 150 mm x 75 um analytical column combined on a single chip. Parameters for the QTOF were as follows: voltage was set at 1750, drying gas 3.5 L/min, temperature 350°C, m/z 300-1800. For MS/MS, the m/z range was 300-2200, and 4 precursors were selected per MS scan. Active exclusion was used after precursors were selected 3 times and exclusion was released after 30 sec. Raw data was extracted and searched using the Spectrum Mill search engine (Rev A.03.03.038 SR1, Agilent Technologies, Palo Alto, CA). “Peak picking” was performed within Spectrum Mill with the following parameters: signal-to-noise was set at 15, a maximum charge state of 4 was allowed (z=4), and the program was directed to attempt to “find” a precursor charge state. Collected spectra were compared to tryptic peptides in the database, and protein identifications were considered significant if the following confidence thresholds were met: minimum of 2 peptides per protein, protein score >10, individual peptide scores of at least 10, and Scored Percent Intensity (SPI) of at least 60%. The SPI provides an indication of the percent of the total ion intensity that matches

the peptide’s MS/MS spectrum. Standards are run at the beginning of each day and at the end of a set of analyses for quality control purposes.

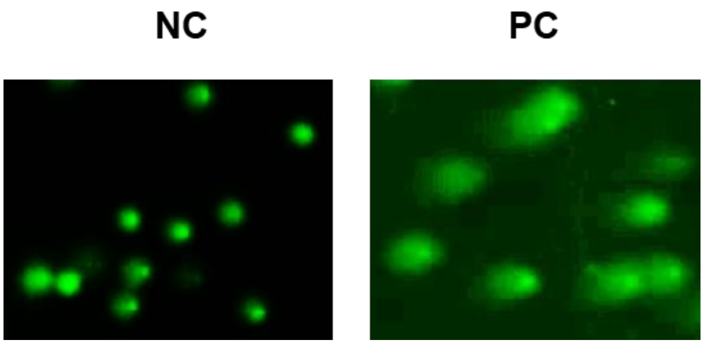

Supplementary Figure S1. Positive control for comet assay to detect DNA damage levels in ATII cells. Freshly isolated ATII cells from non-smokers were used as a negative control (NC) for comet assay. ATII cell treatment with 100 µM H_2O_2 for 1 h was applied as a positive control (PC). Representative images are shown. Magnification 10x40.

A B

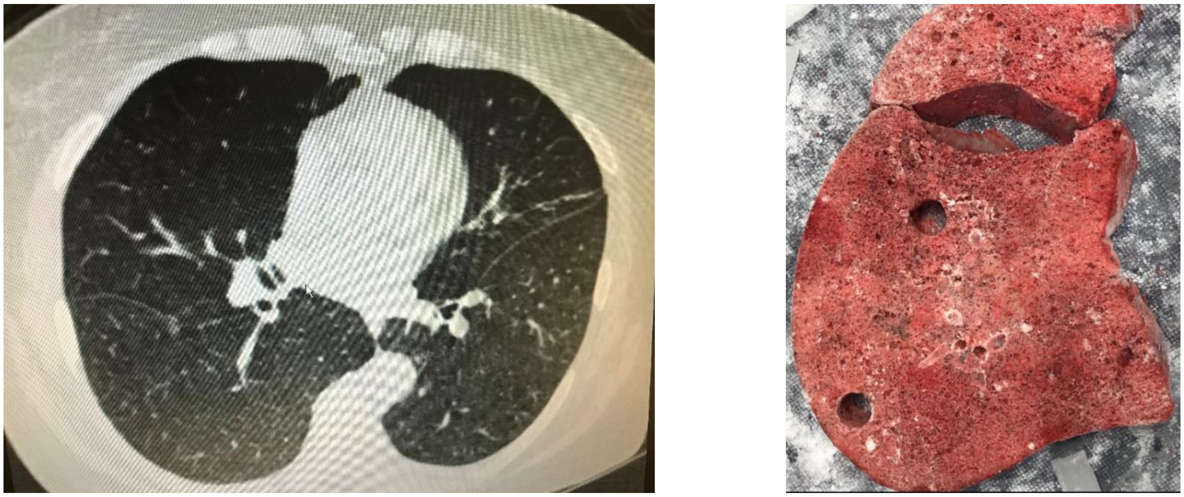

Supplementary Figure S2. Representative CT scan of patient with emphysema. (**A**) Right lung was removed from emphysema patient, inflated with air, frozen in liquid nitrogen vapor and cut into 2 cm high slices from the apex to base of the lung. The slice was obtained from a part of lower and middle lobes, which have both severe and mild emphysema (**B**). Cores were removed from the upper area with severe and lower area with mild emphysema from the same patient. Tissue from cores was used for western blotting to determine protein expression.

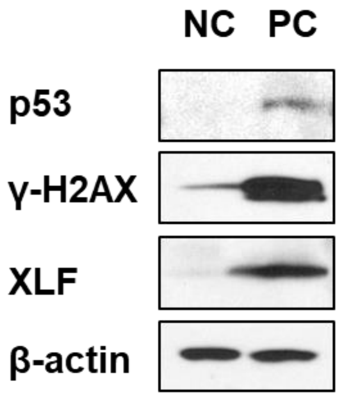

Supplementary Figure S3. Positive control for protein expressions in ATII cells. Freshly isolated ATII cells from non-smokers were used as a negative control (NC). ATII cell treatment with 250nm UV for 5 min and 3 h post-incubation time was applied as a positive control (PC). Representative images of Western blotting are shown.

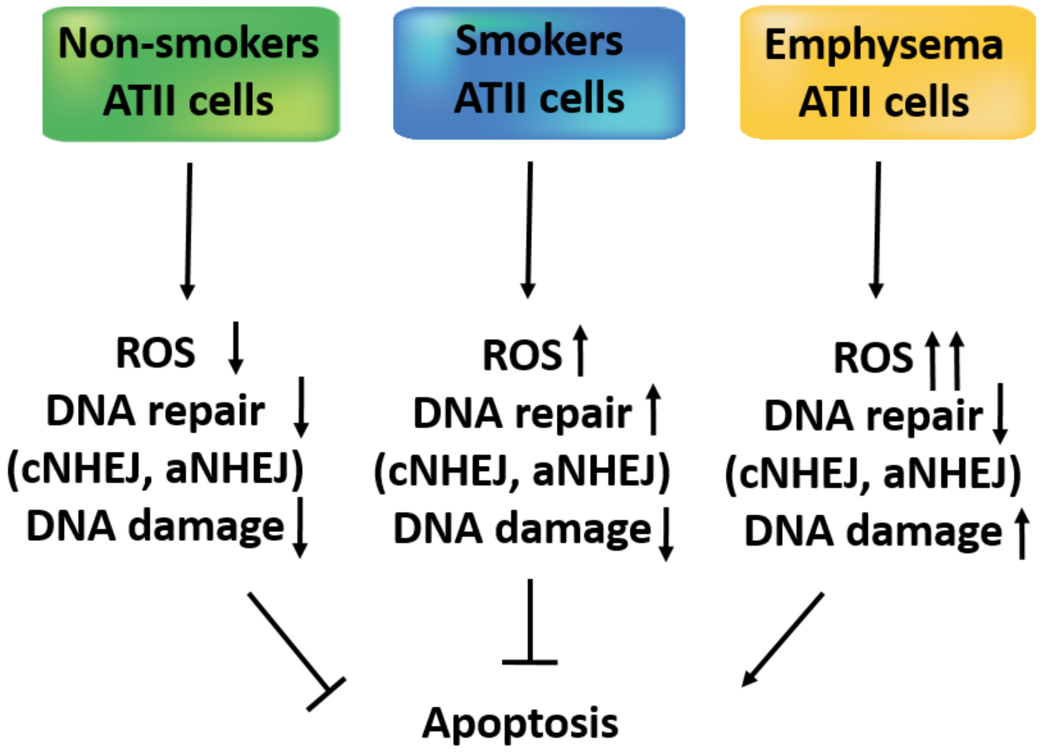

Supplementary Figure S4. Model of ATII cell injury in emphysema. Classical NHEJ (cNHEJ) pathway involves 53BP1, DNA ligase IV, XRCC4 and XLF. Alternative NHEJ (aNHEJ) pathway involves PARP1 and DNA ligase III.
